# Torque differences due to the material variation of the orthodontic appliance: a finite element study

**DOI:** 10.1186/s40510-017-0161-5

**Published:** 2017-02-27

**Authors:** Spyridon N. Papageorgiou, Ludger Keilig, Vaska Vandevska-Radunovic, Theodore Eliades, Christoph Bourauel

**Affiliations:** 1 Clinic of Orthodontics and Pediatric Dentistry, Center of Dental Medicine, University of Zurich, Plattenstrasse 11, 8032 Zurich, Switzerland; 2 Department of Oral Technology, School of Dentistry, University of Bonn, Welschnonnenstr. 17, 53111 Bonn, Germany; 3 Department of Prosthetic Dentistry, Preclinical Education and Materials Science, School of Dentistry, University of Bonn, Bonn, Germany; 4 Department of Orthodontics, Institute of Clinical Dentistry, University of Oslo, Oslo, Norway

**Keywords:** Orthodontics, Fixed appliances, Tooth movement, Torque, Treatment efficiency, Orthodontic materials, Finite element method

## Abstract

**Background:**

Torque of the maxillary incisors is crucial to occlusal relationship and esthetics and can be influenced by many factors. The aim of this study was to assess the relative influence of the material of the orthodontic appliance (adhesive, bracket, ligature, and wire) on tooth displacements and developed stresses/strains after torque application.

**Methods:**

A three-dimensional upper right central incisor with its periodontal ligament (PDL) and alveolus was modeled. A 0.018-in. slot discovery® (Dentaurum, Ispringen, Germany) bracket with a rectangular 0.018 x 0.025-in. wire was generated. The orthodontic appliance varied in the material of its components: adhesive (composite resin or resin-modified glass ionomer cement), bracket (titanium, steel, or ceramic), wire (beta-titanium or steel), and ligature (elastomeric or steel). A total of 24 models were generated, and a palatal root torque of 5° was applied. Afterwards, crown and apex displacement, strains in the PDL, and stresses in the bracket were calculated and analyzed.

**Results:**

The labial crown displacement and the palatal root displacement of the tooth were mainly influenced by the material of the wire (up to 150% variation), followed by the material of the bracket (up to 19% variation). The magnitude of strains developed in the PDL was primarily influenced by the material of the wire (up to 127% variation), followed by the material of the bracket (up to 30% variation) and the ligature (up to 13% variation). Finally, stresses developed at the bracket were mainly influenced by the material of the wire (up to 118% variation) and the bracket (up to 59% variation).

**Conclusions:**

The material properties of the orthodontic appliance and all its components should be considered during torque application. However, these in silico results need to be validated in vivo before they can be clinically extrapolated.

**Electronic supplementary material:**

The online version of this article (doi:10.1186/s40510-017-0161-5) contains supplementary material, which is available to authorized users.

## Background

Tooth inclination in the buccolingual dimension is crucial to the attainment of proper occlusal relationships during treatment and their stability. Improper buccolingual inclinations of the anterior teeth might lead to space deprivation within the dental arch [1], inability to set a solid class I relationship with anterior guidance, and suboptimal smile esthetics, while improper inclinations of the posterior segments might be an obstacle to ideal cusp-to-fossa relationships between the maxillary and mandibular teeth [2]. Therefore, factors that can influence torque like irregularities in tooth anatomy, the size, morphology, and engagement of the archwire in the bracket, as well as the position, slot size, and material properties of the bracket [3–10], need to be taken into account in order to finish optimally the case with effective torque expression that will move the tooth in its proper position in the three planes.

The basis for orthodontic tooth movement is founded in the ability of the periodontal ligament (PDL) and surrounding bone to react to a mechanical stimulus and subsequent displacement of the tooth with remodeling processes [11, 12]. Previous studies have shown that the magnitude of applied forces and of stresses/strains developed in the PDL are associated with the distribution/activity of osteoclasts in it [12, 13] and might be associated with a shift from physiologic to detrimental remodeling phenomena [14, 15], including external apical root resorption. Therefore, as there are indications that torque application is considered a risk factor for external apical root resorption [16, 17], careful monitoring of the biomechanical systems during torque application is warranted.

Complex biomechanical questions like those of orthodontic force application to teeth can be assessed with the finite element (FE) method, as has been done in several cases in order to assess the center of resistance of teeth [18–20], aspects of orthodontic efficiency [21, 22], different bracket [9, 23], anchorage [24, 25] or surgical [26] modalities, and retention procedures [27].

The objective of the present in silico study was to assess the influence of the material characteristics of orthodontic appliances (adhesive, bracket, ligature, and wire) on the biomechanics of torque application. The set-up is similar to a previous study that investigated how differences in the tooth morphology, bracket prescription, and bracket positioning can affect tooth movement after torque application [28].

## Methods

**Fig. 1:**
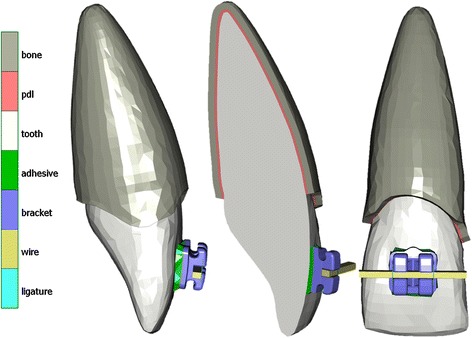
Details of the constructed model with cortical bone layer, periodontal ligament, tooth, adhesive layer, bracket, wire, and ligatures. The outer bone surface was held (boundary condition: fixed nodes in all three axes)

**Fig. 2:**
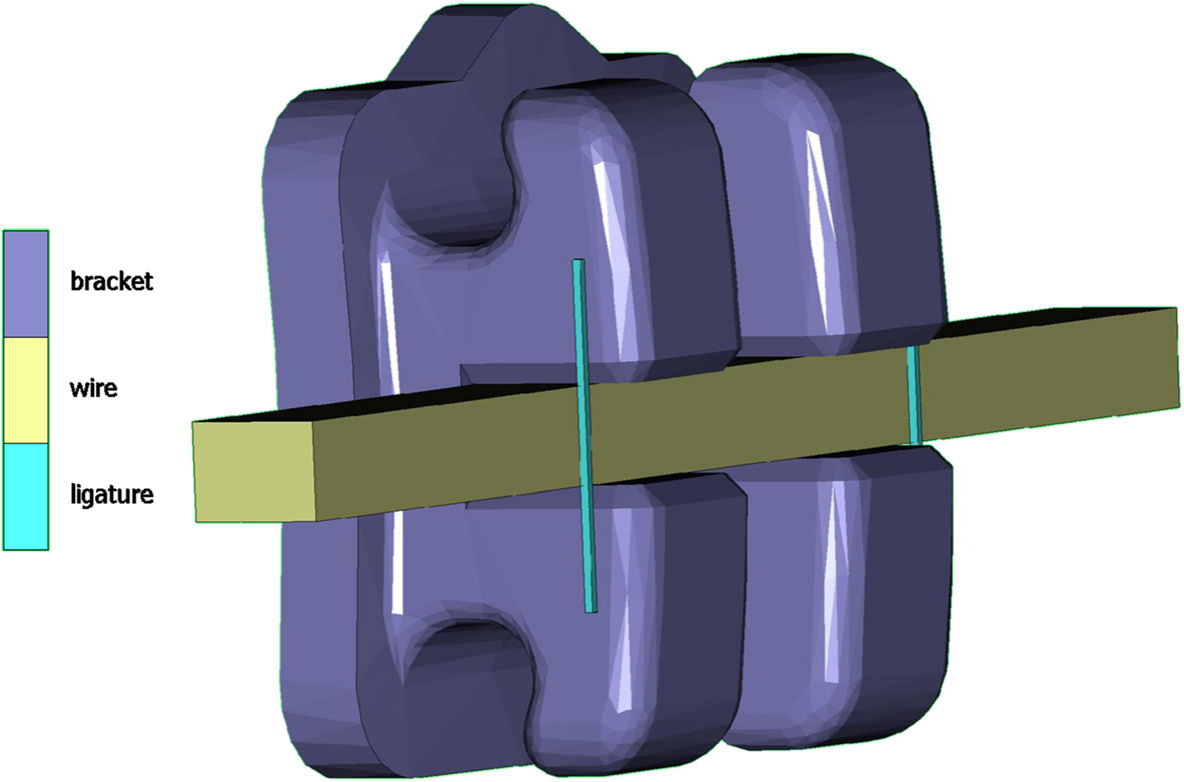
Details of the bracket, wire, and ligatures

A three-dimensional (3D) solid model was constructed including a maxillary right central incisor with its PDL and alveolus and a uniform thickness of 0.2 and 0.5 mm, respectively (Fig. 1). The base geometry of the tooth model was derived from a commercial three-dimensional dataset, based on a larger survey of Caucasian patients (“teeth with roots and gum”, Viewpoint Data Labs, now Digimation Inc., Lake Mary, FL, USA) with an average crown-to-root inclination [29]. A partial orthodontic fixed appliance was constructed with a composite resin adhesive layer (mean thickness 0.2 mm) and a bracket at the center of the labial incisor crown surface, while a rectangular 0.46 x 0.64-mm (0.018 x 0.025 in.) wire was passively inserted into the bracket slot and ligated with two ligatures (Fig. 2). For all models, the same Standard Edgewise (0° torque prescription) bracket was used, based on computer-aided design and computer-aided manufacturing (CAD/CAM) data of the discovery® bracket (Dentaurum, Ispringen, Germany), provided by the manufacturer in 0.46-mm (0.018 in.) slot, as this is more efficient in torque expression with a slot-filling archwire than the 0.56-mm (0.022 in.) slot [30].

Based on these 3D solid models, an FE mesh was created to make a node-to-node connection between the bracket, adhesive, tooth, PDL, and alveolar bone with a coarsening factor of 1.5, which was previously seen to be reliable [18]. An FE mesh of the wire and the ligatures was created separately from the bracket to allow contact analyses based on the Coulomb friction model in the FE program used (MSC.Marc/Mentat v. 2010, MSC Software Corp., Santa Ana, CA, USA) with a bracket-wire frictional coefficient of 0.2. The 3D FE model consisted of 68,023 isoparametric tetrahedral solid elements (four noded).

The materials tested in this study pertained to the adhesive (composite resin or resin-modified glass ionomer cement), the bracket (titanium, stainless steel, or ceramic), the wire (beta-titanium or stainless steel), and the ligature (elastomeric or stainless steel). The material properties used in this study were based on previously published studies (Table 1) [9]. All materials were considered to be homogenous and isotropic apart from the PDL, which was modeled as bilinear elastic (*E*_1_ = 0.05 MPa; *E*_2_ = 0.20 MPa; *ε*_12_ = 7%) [19].

**Table 1:** Material properties used in this study

Material	Young’s modulus (MPa)	Poisson’s ratio
Bone	2000	0.30
PDL	bilinear: 0.05/0.20 ultimate strain, *ε*_12_ 7.0%	0.30
Tooth	20,000	0.30
Adhesive—composite resin	8823	0.25
Adhesive—RMGI	7600	0.30
Bracket—titanium	114,000	0.30
Bracket and ligatures—stainless steel	200,000	0.30
Bracket—ceramic	379,000	0.29
Wire—TMA	65,000	0.30
Wire—stainless steel	200,000	0.30
Ligature—elastomeric	100	0.30

*PDL* periodontal ligament, *TMA* titanium molybdenum alloy, *RMGI* resin-modified glass ionomer cement

The simulation was designed to reflect the clinical situation of an active palatal root torque of 5° acting from the twisted wire on an incisor. The wire was inserted passively on the bottom of the bracket slot prior to torque application. The boundary conditions included holding the apical bone surface (movement restriction of outer bone surface) and keeping the ligatures tight with a spring nodal tie, while torque was applied at the two ends of the wire. The induced palatal movement of the root tip, labial movement of the crown tip, total equivalent strains in the PDL, and the von Mises stresses in the bracket were calculated at the simulation’s end as the maximum value within the volume of the corresponding body. Mean values across models according to the various parameters were calculated and analyzed descriptively. All simulations were performed with the abovementioned FE software (convergence tolerance for residual relative force = 0.1 and convergence tolerance for the incremental rotations of rigid link nodes = 0.001). Models were created on a Dell Precision T5500 workstation (Dell, Frankfurt, Germany) and transferred to a 30-processor Dell server cluster to be solved. A sensitivity analysis to check the reliability of the existing mesh was performed by subdividing all elements across all three dimensions of a randomly chosen model, thereby effectively octupling the total number of elements in the model.

## Results

**Fig. 3:**
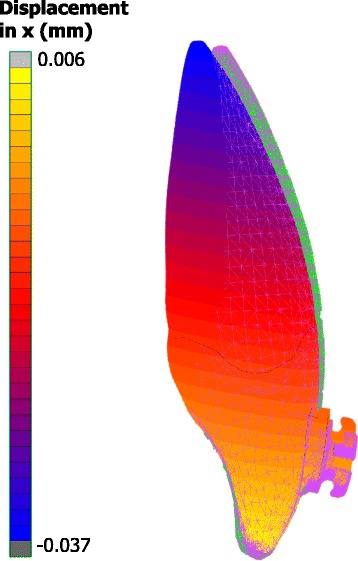
Example showing the distribution of calculated displacements in the labiolingual direction. Displacements are magnified optically by 30 at the last increment (tooth with contour bands) to differentiate it from the initial model (tooth with *green* mesh)

**Fig. 4:**
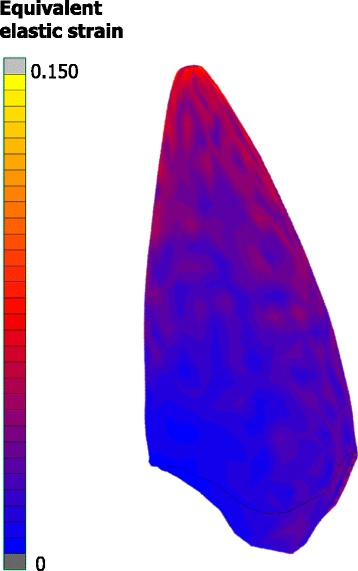
Example showing the distribution of equivalent elastic strain in the periodontal ligament

**Fig. 5:**
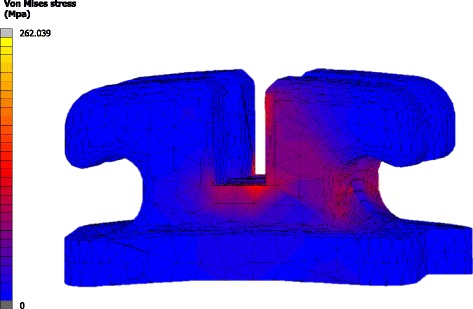
Example showing the distribution of von Mises stresses in the bracket

Characteristic examples of the analysis results are illustrated in Figs. 3, 4, and 5. In all cases, the crown tip was displaced labially and the root tip was displaced palatally. Developed strains in the PDL were mostly distributed at the apical regions, where root tip was displaced. Conversely, stresses at the bracket were mostly concentrated at the bracket wall, where the wire’s edge came into contact with the bracket. The effect of the material of the adhesive, bracket, wire, and ligatures on tooth displacement, developed strains in the PDL, and developed stresses in the bracket can be seen in full in Additional file 1, in Table 2 as absolute change, and in Table 3 as percentage change.

**Table 2:** Absolute changes in the various models according to the material of each component

Factor	Group	Absolute crown displacement (mm)	Absolute apex displacement (mm)	Equivalent elastic strain in PDL	Von Mises stress in bracket (MPa)
Adhesive	RMGI	Ref (0.0102)	Ref (0.0513)	Ref (0.2405)	Ref (397.1245)
	Composite resin	+0.0000	+0.0001	−0.0010	+0.0555
Bracket	Titanium	Ref (0.0093)	Ref (0.0501)	Ref (0.2159)	Ref (305.7099)
	Stainless steel	+0.0010	+0.0014	+0.0068	+90.0031
	Ceramic	+0.0018	+0.0021	+0.0655	+181.3240
Ligature	Elastomeric	Ref (0.0100)	Ref (0.0511)	Ref (0.2565)	Ref (389.9210)
	Stainless steel	+0.0003	+0.0004	−0.0331	+14.4625
Wire	TMA	Ref (0.0058)	Ref (0.0361)	Ref (0.1470)	Ref (249.8552)
	Stainless steel	+0.0087	+0.0304	+0.1859	+294.5942

*PDL* periodontal ligament, *TMA* titanium molybdenum alloy, *RMGI* resin-modified glass ionomer cement, *Ref* reference category consisting of the average across models with this material

**Table 3:** Percentage changes in the various models according to the material of each component

Factor	Group	Absolute crown displacement (mm)	Absolute apex displacement (mm)	Equivalent elastic strain in PDL	Von Mises stress in bracket (MPa)
Adhesive	RMGI	Ref (100%)	Ref (100%)	Ref (100%)	Ref (100%)
	Composite resin	+0%	+0.2%	−0.4%	+0%
Bracket	Titanium	Ref (100%)	Ref (100%)	Ref (100%)	Ref (100%)
	Stainless steel	+10.8%	+2.8%	+3.1%	+29.4%
	Ceramic	+18.8%	+4.2%	+30.3%	+59.3%
Ligature	Elastomeric	Ref (100%)	Ref (100%)	Ref (100%)	Ref (100%)
	Stainless steel	+3.0%	+0.8%	−12.9%	+3.7%
Wire	TMA	Ref (100%)	Ref (100%)	Ref (100%)	Ref (100%)
	Stainless steel	+150.0%	+84.2%	+126.5%	+117.9%

*PDL* periodontal ligament, *TMA* titanium molybdenum alloy, *RMGI* resin-modified glass ionomer cement, *Ref* reference category consisting of the average across models with this material

The average across models of the maximum labial displacement of the crown tip was 0.010 mm. There was a miniscule influence by the ligature material (up to 3%), a small influence from the bracket material (up to 19%), and a large influence from the wire material (up to 150%), where stainless steel wires were associated with greater displacement.

The average across models of the maximum palatal displacement of the root apex was 0.051 mm. There was only a small influence by the bracket material (up to 4%) and a large influence from the wire material (up to 84%), where stainless steel wires were associated with greater displacement.

The average across models of the maximum strains in the PDL was 0.240 with a small influence from the material of the ligature (up to 13%) and the bracket (up to 30%) and a large influence from the wire material (up to 127%). As expected, torque application with stainless steel wires was associated with increased strains in the PDL.

Finally, the average across models of the maximum induced stresses in the bracket was 397.2 MPa. Here, the material of the adhesive or the ligature did not have a considerable effect, while the material of the bracket had a moderate influence (up to 59%) and the material of the bracket itself had a large influence (up to 118%).

The performed sensitivity analysis (Table 4) indicated that the sharp increase in the total number of elements did not have a considerable influence on the results, as all deviations were in the level of 7–14% for the majority, which is in the range of FE analyses in general.

**Table 4:** Sensitivity analysis by octupling the total number of elements in the model

	Original	Sensitivity (×8)		
	Elements	Elements		
Adhesive	2351	18,808		
Bone	6835	54,680		
Bracket	28,791	230,328		
Left ligature	597	4776		
Right ligature	597	4776		
PDL	5777	46,216		
Tooth	10,518	84,144		
Wire	12,557	100,456		
	Absolute crown displacement (mm)	Absolute apex displacement (mm)	Equivalent elastic strain in PDL	Von Mises stress in bracket (MPa)
Original	0.006	0.036	0.146	237.975
Sensitivity	0.004	0.031	0.156	297.663
Difference	−0.002	−0.005	+0.010	+21.756
Difference %	−33.3%	−13.9%	+6.8%	+9.1%

*PDL* periodontal ligament

## Discussion

In this study, the relative contribution of the material variation for the adhesive, the bracket, the wire, and the ligature to the attained tooth displacement and the developed von Mises stresses and strains in the PDL and the bracket were investigated in silico. The von Mises stresses are often used in finite element analyses due to their efficacy, as they allow for the combination of principal stresses into an equivalent stress that is comparable to the yield stress, hence giving a better chance of determining the failure of the system. It was observed that the displacement of the crown and the root were mainly affected by the material of the wire, the bracket, and the ligature. The strains induced at the PDL level were affected mainly by the wire material, with only minor influences of the adhesive, the bracket, and the ligature. Finally, the material of the bracket and the wire had a considerable effect on the developed stresses at the bracket level during torque application.

The finite element method enables us to answer complex biomechanical questions in the field of orthodontics via simulation; moreover, it enables investigators to predict the behavior of biological structures in many specific situations. However, any solutions obtained via FE simulation will be numerical approximations. Although many measurements cannot be taken in vivo, they can nevertheless contribute useful information to clinical investigations.

Orthodontically induced root resorption is a multifaceted phenomenon with complex etiopathology. Although the duration of treatment with heavy rectangular wires and especially excessive torque application might be regarded as risk factors, no single mechanical factor can fully predict treatment-induced resorption of the root. An additional detrimental factor for the development of root resorption might be the iatrogenic approximation of the anterior tooth roots towards the cortical plate, which has been found to be significantly associated with the amount of resorption [16, 31]. This might play a role, since existing data indicate that considerable variation exists in the alveolar thickness buccal and lingual of the upper incisors according to tooth type and facial type [32]. In the present study, only the material of the wire had a considerable effect on the palatal displacement of the root tip. Additionally, a previous study has shown that bracket prescription and especially bracket positioning can have a considerable effect on the displacement of the root apex [9]. These factors should be appropriately considered, as labial uprighting of such palatally torqued crowns, with the subsequent larger palatal displacement of the root, might be limited due to anatomical reasons [33].

The strengths of this study include the bilinear modeling of the PDL, which is more accurate than the commonly used simplified linear modeling of the PDL [34, 35]. All material properties used were based on the previous studies. To reduce the systematic error, no absolute values were used to draw any conclusions, and only the differences between the simulations were considered. Since all simulations were affected by the simplification effects to the same extent, the analysis of the differences resulted in an additional increase of validity.

Comparisons with other studies are limited, due to the absence of studies with similar scope and outcomes. There are additional factors that might influence the biomechanical behavior of fixed appliances. Significant differences in the tie-wing tensile fracture strength of semi-twin and true-twin brackets have been reported [36]. Likewise, all brackets modeled consisted from a single material phase, and no different materials were used for the tie-wings and base of the bracket, as is sometimes done for metallic brackets [37].

Several considerations should be taken into account, when interpreting the results of this study. As the scope was to investigate the net effective torque on the tooth and the surrounding structures, full wire-bracket engagement was modeled with idealized bracket and wire dimensions. In reality, smaller wire dimensions, the use of a 0.022-in. bracket system, or the reported dimensional inaccuracy of wires and brackets [2, 38] would most likely introduce additional wire play [21] and thereby decrease effective torque application. The values reported in this study correspond to the moment or root movement variants in cases of play minimization by the use of terminal sized or excessively torqued archwires, which should counteract the play and care should be exercised in transferring the results of this investigation to the clinical situation. Furthermore, the present study assesses relative contributions of various factors to the initial force system applied singularly to an upper central incisor, which might not directly reflect clinical scenarios with full archwire engagement. To reduce the number of equations to be solved, the teeth were not differentiated into enamel, dentine, pulp, and cementum but were provided uniformly with the elasticity parameters of dentine. In view of the minor forces applied, the influence of this simplification is negligible because no substantial deformation of the dental hard tissue was to be expected. For the same reason, the bone was not differentiated into cancellous and cortical bone, while no nickel-titanium wire was modeled, due to its complex mechanical properties. Finally, future studies on this research field should assess the effect of different biomechanical strategies of torque application with the risk and extent of root resorption.

As far as clinical implications are concerned, careful consideration of material choice for the orthodontic appliance is warranted, especially in cases of limited alveolar thickness. Although third-order recommendations for upper incisors seem to be unaffected by the adhesive, the material of the bracket and especially the wire influence directly the tooth displacement and the developed stresses/strains. From a biological point of view, the use of a TMA wire would be favorable over a stainless steel wire in order to reduce the developed strains in the PDL, even though if the latter is more effective in displacing the tooth. Additionally, the use of a ceramic bracket ligated with steel ligatures might be handy in order to maximize the attained labial crown torque. In any case, a common “one-size-fits-all” fully prescribed straight-wire appliance might not be appropriate to every single patient, whereas individualized treatment planning for orthodontic mechanotherapy might be favorable.

## Conclusions

According to this in silico study, the following conclusions can be drawn:

- The magnitude of the displacement of the crown tip or apex seems to be considerably influenced by the material of the wire (up to 112% variation), the bracket (up to 42% variation), and the ligature (up to 7% variation).
- The magnitude of strains developed in the PDL was primarily influenced by the material of the wire (up to 65% variation), followed by the material of the ligature (up to 17% variation), the bracket (up to 12% variation), and the adhesive (up to 13% variation).
- The stresses developed within the bracket seem to be mainly influenced by the material of the bracket (up to 116% variation) and the wire (up to 56% variation).

As a result, these factors should be taken into consideration for each separate case, and the careful consideration of the orthodontic appliance used is warranted, when applying torque on upper incisors. However, clinical studies are needed to verify these findings.

## Additional file

Additional file 1: Simulation results for the constructed models at the last increment of the 5° torque application. Shown are the maximum values for each calculated outcome within the corresponding body volume. (DOC 59 kb)

## References

[1] O’Higgins EA, Kirschen RH, Lee RT (1999). The influence of maxillary incisor inclination on arch length. Br J Orthod.

[2] Gioka C, Eliades T (2004). Materials-induced variation in the torque expression of preadjusted appliances. Am J Orthod Dentofacial Orthop.

[3] Sebanc J, Brantley WA, Pincsak JJ, Conover JP (1984). Variability of effective torque as a function of edge bevel on orthodontic arch wires. Am J Orthod.

[4] Germane N, Bentley BE, Isaacson RJ (1989). Three biologic variables modifying faciolingual tooth angulation by straight-wire appliances. Am J Orthod Dentofacial Orthop.

[5] Miethke RR, Melsen B (1999). Effect of variation in tooth morphology and bracket position on first and third order correction with preadjusted appliances. Am J Orthod.

[6] Morina E, Eliades T, Pandis N, Jäger A, Bourauel C (2008). Torque expression of self-ligating brackets compared with conventional metallic, ceramic, and plastic brackets. Eur J Orthod.

[7] Archambault A, Major TW, Carey JP, Heo G, Badawi H, Major PW (2010). A comparison of torque expression between stainless steel, titanium molybdenum alloy, and copper nickel titanium wires in metallic self-ligating brackets. Angle Orthod.

[8] Papageorgiou SN, Konstantinidis I, Papadopoulou K, Jäger A, Bourauel C (2014). Clinical effects of pre-adjusted edgewise orthodontic brackets: a systematic review and meta-analysis. Eur J Orthod.

[9] Papageorgiou SN, Keilig L, Hasan I, Jäger A, Bourauel C (2016). Effect of material variation on the biomechanical behaviour of orthodontic fixed appliances: a finite element analysis. Eur J Orthod.

[10] Papageorgiou SN, Gölz L, Jäger A, Eliades T, Bourauel C (2016). Lingual vs. labial fixed orthodontic appliances: systematic review and meta-analysis of treatment effects. Eur J Oral Sci.

[11] Turner CH, Pavalko FM (1998). Mechanotransduction and functional response of the skeleton to physical stress: the mechanisms and mechanics of bone adaptation. J Orthop Sci.

[12] Melsen B (2001). Tissue reaction to orthodontic tooth movement—a new paradigm. Eur J Orthod.

[13] Kawarizadeh A, Bourauel C, Zhang D, Götz W, Jäger A (2004). Correlation of stress and strain profiles and the distribution of osteoclastic cells induced by orthodontic loading in rat. Eur J Oral Sci.

[14] Khouw FE, Goldhaber P (1970). Changes in vasculature of the periodontium associated with tooth movement in the rhesus monkey and dog. Arch Oral Biol.

[15] Quinn RS, Yoshikawa DK (1985). A reassessment of force magnitude in orthodontics. Am J Orthod.

[16] Kaley J, Phillips C (1991). Factors related to root resorption in edgewise practice. Angle Orthod.

[17] Weltman B, Vig KW, Fields HW, Shanker S, Kaizar EE (2010). Root resorption associated with orthodontic tooth movement: a systematic review. Am J Orthod Dentofacial Orthop.

[18] Reimann S, Keilig L, Jäger A, Bourauel C (2007). Biomechanical finite-element investigation of the position of the centre of resistance of the upper incisors. Eur J Orthod.

[19] Kettenbeil A, Reimann S, Reichert C, Keilig L, Jäger A, Bourauel C (2013). Numerical simulation and biomechanical analysis of an orthodontically treated periodontally damaged dentition. J Orofac Orthop.

[20] Viecilli RF, Budiman A, Burstone CJ (2013). Axes of resistance for tooth movement: does the center of resistance exist in 3-dimensional space?. Am J Orthod Dentofacial Orthop.

[21] Tominaga JY, Chiang PC, Ozaki H, Tanaka M, Koga Y, Bourauel C, Yoshida N (2012). Effect of play between bracket and archwire on anterior tooth movement in sliding mechanics: a three-dimensional finite element study. J Dent Biomech.

[22] Tominaga JY, Ozaki H, Chiang PC, Sumi M, Tanaka M, Koga Y, Bourauel C, Yoshida N (2014). Effect of bracket slot and archwire dimensions on anterior tooth movement during space closure in sliding mechanics: a 3-dimensional finite element study. Am J Orthod Dentofacial Orthop.

[23] Huang Y, Keilig L, Rahimi A, Reimann S, Eliades T, Jäger A, Bourauel C (2009). Numeric modeling of torque capabilities of self-ligating and conventional brackets. Am J Orthod Dentofacial Orthop.

[24] Reimann S, Keilig L, Jäger A, Brosh T, Shpinko Y, Vardimon AD, Bourauel C (2009). Numerical and clinical study of the biomechanical behaviour of teeth under orthodontic loading using a headgear appliance. Med Eng Phys.

[25] Chatzigianni A, Keilig L, Duschner H, Götz H, Eliades T, Bourauel C (2011). Comparative analysis of numerical and experimental data of orthodontic mini-implants. Eur J Orthod.

[26] MacGinnis M, Chu H, Youssef G, Wu KW, Machado AW, Moon W (2014). The effects of micro-implant assisted rapid palatal expansion (MARPE) on the nasomaxillary complex—a finite element method (FEM) analysis. Prog Orthod.

[27] Jahanbin A, Abtahi M, Heravi F, Hoseini M, Shafaee H (2014). Analysis of different positions of fiber-reinforced composite retainers versus multistrand wire retainers using the finite element method. Int J Biomater.

[28] Papageorgiou SN, Sifakakis I, Keilig L, Patcas R, Affolter S, Eliades T, Bourauel C. Torque differences according to tooth morphology and bracket placement: a finite element study. Eur J Orthod 2016 [Epub ahead of print] 10.1093/ejo/cjw074 27932407

[29] Delivanis HP, Kuftinec MM (1980). Variation in morphology of the maxillary central incisors found in Class II, Division 2 malocclusions. Am J Orthod.

[30] Papageorgiou SN, Sifakakis I, Doulis I, Eliades T, Bourauel C (2016). Torque efficiency of square and rectangular archwires into 0.018 and 0.022 in. conventional brackets. Prog Orthod.

[31] Horiuchi A, Hotokezaka H, Kobayashi K (1998). Correlation between cortical plate proximity and apical root resorption. Am J Orthod Dentofacial Orthop.

[32] Gracco A, Lombardo L, Mancuso G, Gravina V, Siciliani G (2009). Upper incisor position and bony support in untreated patients as seen on CBCT. Angle Orthod.

[33] Harris EF, Hassankiadeh S, Harris JT (1993). Maxillary incisor crown-root relationships in different angle malocclusions. Am J Orthod Dentofacial Orthop.

[34] Ziegler A, Keilig L, Kawarizadeh A, Jäger A, Bourauel C (2005). Numerical simulation of the biomechanical behaviour of multi-rooted teeth. Eur J Orthod.

[35] Dong-Xu L, Hong-Ning W, Chun-Ling W, Hong L, Ping S, Xiao Y (2011). Modulus of elasticity of human periodontal ligament by optical measurement and numerical simulation. Angle Orthod.

[36] Johnson G, Walker MP, Kula K (2005). Fracture strength of ceramic bracket tie wings subjected to tension. Angle Orthod.

[37] Zinelis S, Annousaki O, Eliades T, Makou M (2004). Elemental composition of brazing alloys in metallic orthodontic brackets. Angle Orthod.

[38] Joch A, Pichelmayer M, Weiland F (2010). Bracket slot and archwire dimensions: manufacturing precision and third order clearance. J Orthod.

