# Supplementary material for: Torque differences due to the material variation of the orthodontic appliance: a finite element study
Source: Prog Orthod. 2017 Feb 27;18:6. doi: 10.1186/s40510-017-0161-5 (PMC5326743; doi:10.1186/s40510-017-0161-5)
Supplement: Additional file 1: — Simulation results for the constructed models at the last increment of the 5° torque application. Shown are the maximum values for each calculated outcome within the corresponding body volume. (DOC 59 kb) [file 40510_2017_161_MOESM1_ESM.doc]

**Torque differences due to the material variation of the orthodontic appliance: a finite element study**

**Supplementary material**

**Supplement**. Simulation results for the constructed models at the last increment of the 5 degree torque application. Shown are the maximum values for each calculated outcome within the corresponding body volume.

| Model | Adhesive | Bracket | Ligature | Wire | Absolute crown displacement (mm) | Absolute apex displacement (mm) | Equivalent elastic strain in PDL | Von Mises stress in bracket (MPa) |
| --- | --- | --- | --- | --- | --- | --- | --- | --- |
| 1 | RMGI | Ti | Elast. | TMA | 0.005 | 0.035 | 0.144 | 218.178 |
| 2 | RMGI | SS | Elast. | TMA | 0.006 | 0.036 | 0.146 | 237.930 |
| 3 | RMGI | CER | Elast. | TMA | 0.006 | 0.036 | 0.147 | 256.691 |
| 4 | RMGI | Ti | SS | TMA | 0.006 | 0.036 | 0.146 | 243.702 |
| 5 | RMGI | SS | SS | TMA | 0.006 | 0.037 | 0.149 | 262.008 |
| 6 | RMGI | CER | SS | TMA | 0.006 | 0.036 | 0.149 | 280.543 |
| 7 | RMGI | Ti | Elast. | SS | 0.013 | 0.065 | 0.287 | 380.949 |
| 8 | RMGI | SS | Elast. | SS | 0.014 | 0.066 | 0.295 | 578.729 |
| 9 | RMGI | CER | Elast. | SS | 0.016 | 0.068 | 0.527 | 666.668 |
| 10 | RMGI | Ti | SS | SS | 0.013 | 0.064 | 0.286 | 380.193 |
| 11 | RMGI | SS | SS | SS | 0.015 | 0.067 | 0.300 | 515.858 |
| 12 | RMGI | CER | SS | SS | 0.016 | 0.069 | 0.310 | 744.045 |
| 13 | CR | Ti | Elast. | TMA | 0.005 | 0.036 | 0.145 | 218.202 |
| 14 | CR | SS | Elast. | TMA | 0.006 | 0.036 | 0.146 | 237.975 |
| 15 | CR | CER | Elast. | TMA | 0.006 | 0.036 | 0.147 | 256.717 |
| 16 | CR | Ti | SS | TMA | 0.006 | 0.036 | 0.146 | 243.713 |
| 17 | CR | SS | SS | TMA | 0.006 | 0.037 | 0.150 | 262.039 |
| 18 | CR | CER | SS | TMA | 0.006 | 0.036 | 0.149 | 280.564 |
| 19 | CR | Ti | Elast. | SS | 0.013 | 0.065 | 0.287 | 381.069 |
| 20 | CR | SS | Elast. | SS | 0.014 | 0.066 | 0.295 | 579.088 |
| 21 | CR | CER | Elast. | SS | 0.016 | 0.068 | 0.512 | 666.856 |
| 22 | CR | Ti | SS | SS | 0.013 | 0.064 | 0.286 | 379.673 |
| 23 | CR | SS | SS | SS | 0.015 | 0.067 | 0.300 | 516.077 |
| 24 | CR | CER | SS | SS | 0.016 | 0.069 | 0.310 | 744.187 |
|  |  |  |  |  |  |  |  |  |
|  |  |  |  | Minimum | 0.005 | 0.035 | 0.144 | 218.178 |
|  |  |  |  | Average | 0.010 | 0.051 | 0.240 | 397.152 |
|  |  |  |  | Maximum | 0.016 | 0.069 | 0.527 | 744.187 |

PDL, periodontal ligament; RMGI, resin modified glass ionomer cement; CR, composite resin; Ti, titanium alloy; SS, stainless steel; CER, ceramic; Elast., elastometic; TMA, titanium molybdenum alloy.
